# Estrategia de apropiación social del conocimiento en la isquemia cardiaca*

**DOI:** 10.15649/cuidarte.2823

**Published:** 2023-09-08

**Authors:** Diana Marcela Castillo Sierra, Lina María Vargas-Escobar, Melany Johana Moreno Nieto, Sandra Patricia Díaz Farfán, Karen Tatiana Rodríguez Marín

**Affiliations:** 1 Facultad de Enfermería-Universidad El Bosque, Bogotá, Colombia. E-mail. dmcastillos@unbosque.edu.co Universidad El Bosque Facultad de Enfermería Universidad El Bosque Bogotá Colombia dmcastillos@unbosque.edu.co; 2 Facultad de Enfermería-Universidad El Bosque, Bogotá, Colombia. E-mail: lmvargase@unbosque.edu.co Universidad El Bosque Facultad de Enfermería Universidad El Bosque Bogotá Colombia lmvargase@unbosque.edu.co; 3 Facultad de Enfermería-Universidad El Bosque, Bogotá, Colombia. E-mail: mjmoreno@unbosque.edu.co Universidad El Bosque Facultad de Enfermería Universidad El Bosque Bogotá Colombia mjmoreno@unbosque.edu.co; 4 Facultad de Enfermería-Universidad El Bosque, Bogotá, Colombia. E-mail: sdiazf@unbosque.edu.co Universidad El Bosque Facultad de Enfermería Universidad El Bosque Bogotá Colombia sdiazf@unbosque.edu.co; 5 Facultad de Enfermería-Universidad El Bosque, Bogotá, Colombia. E-mail. ktrodriguezm@unbosque.edu.co Universidad El Bosque Facultad de Enfermería Universidad El Bosque Bogotá Colombia ktrodriguezm@unbosque.edu.co

**Keywords:** Educación en Salud, Enfermedades Cardiovasculares, Isquemia Miocárdica, Enfermería Cardiovascular, Estrategia de Salud Digital, Health Education, Cardiovascular Diseases, Myocardial Ischemia, Cardiovascular Nursing, Health Strategies, Educagáo em Saúde, Doengas Cardiovasculares, Isquemia Miocárdica, Enfermagem Cardiovascular, Estratégias de Saúde

## Abstract

**Introducción:**

La construcción de conocimiento a partir del intercambio de saberes entre pacientes, familias y equipo de salud es una necesidad sentida que puede impactar en la educación en salud y la adherencia a los tratamientos.

**Objetivo:**

Desarrollar una estrategia de apropiación social que permita innovar en el cuidado de la salud de las personas con enfermedad isquémica cardiaca y sus familias mediante el uso de la herramienta MHealth.

**Materiales y Métodos:**

Estudio exploratorio que permitió el diseño de una estrategia de apropiación social de conocimiento para mejorar la salud cardiovascular de personas que sufren isquemia cardiaca. Participaron 31 personas entre pacientes, familiares, profesionales de la salud. Se desarrollaron dos talleres virtuales en el mes de junio y seguimientos telefónicos entre agosto y septiembre. Las experiencias y saberes de los participantes, permitió la construcción de la herramienta MHealth.

**Resultados:**

La reflexión crítica entre los actores facilitó la construcción y desarrollo de la herramienta tipo MHealth (página web/app) titulada “Cuidando tu corazón”, que permite el acceso a información relacionada con el cuidado durante la isquemia cardiaca y articula información a partir del diálogo de saberes y la evidencia reportada en la literatura.

**Discusión:**

Las estrategias de apropiación social son una herramienta relevante para la generación de conocimiento en el área de la salud.

**Conclusiones:**

La estrategia implementada permitió reconocer la importancia de contar con herramientas para el cuidado de la salud cardiovascular elaboradas desde las necesidades, perspectivas y creencias de sus actores para mejorar la educación y el cuidado.

## Abstract (en)

**Introduction:**

The construction of knowledge from the exchange of knowledge between patients, families, and healthcare teams is a felt need that can impact health education and adherence to treatment.

**Objective:**

To develop a social appropriation strategy that enables innovation in the health care of people with ischemic heart disease and their families through the use of an MHealth tool.

**Materials and methods:**

Exploratory study that allowed the design of a strategy for the social appropriation of knowledge to improve the cardiovascular health of people suffering from myocardial ischemia. Thirty-one people participated, including patients, family members, and health professionals. Two virtual workshops were held in June, and follow-up phone calls were made between August and September. The participant's experiences and knowledge enabled the construction of the MHealth tool.

**Results:**

The critical reflection of the stakeholders facilitated the construction and development of the MHealth tool (webpage/app) named “Cuidando tu corazón” (Taking care of your heart).

**Discussion:**

Social appropriation strategies are a relevant tool for knowledge creation in health.

**Conclusions:**

The strategy implemented made it possible to recognize the importance of developing cardiovascular healthcare tools from stakeholders' needs, perspectives, and beliefs to improve education and care.

## Resumo

**Introdujo:**

A construyo do conhecimento a partir da troca de conhecimentos entre pacientes, familiares e equipe de saúde é uma necessidade sentida que pode impactar na educagáo em saúde e na adesáo aos tratamentos.

**Objetivo:**

Desenvolver uma estratégia de apropriagáo social que permita inovar na atengáo a saúde de pessoas com doenga isquémica do coragáo e seus familiares por meio do uso da ferramenta MHealth.

**Materiais e Métodos:**

Estudo exploratório que permitiu desenhar uma estratégia de apropriagáo social do conhecimento para melhorar a saúde cardiovascular de pessoas com isquemia cardíaca. Participaram 31 pessoas, entre pacientes, familiares e profissionais de saúde. Foram realizadas dois seminários virtuais em junho e acompanhamentos por telefone entre agosto e setembro. As experiencias e conhecimentos dos participantes permitiram a construyo da ferramenta MHealth.

**Resultados:**

A reflexáo crítica entre os atores facilitou a construyo e desenvolvimento da ferramenta do tipo MHealth (página web/ aplicativo) intitulada "Cuidando do seu coragáo", que permite o acesso as informales relacionadas aos cuidados durante a isquemia cardíaca e articula as informales a partir do diálogo de conhecimento e as evidencias relatadas na literatura.

**Discussao:**

As estratégias de apropriagáo social sáo uma ferramenta relevante para a geragáo de conhecimento na área da saúde.

**Condusdes:**

A estratégia implementada possibilitou reconhecer a importancia de ter ferramentas para o cuidado a saúde cardiovascular elaboradas a partir das necessidades, perspectivas e crengas de seus atores para melhorar a educagáo e o cuidado.

## Introducción

Las enfermedades cardiovasculares (ECV) han sido la principal causa de muerte en el mundo durante los últimos 15 años. El estudio Global Burden of Disease estimó en el 2013 que la enfermedad cardiovascular provocó 17.3 millones de muertes, es decir, el 31.5% de todas las defunciones. Así mismo, se ha descrito que los adultos de países de bajos y medianos ingresos tienen un mayor riesgo de mortalidad que en los países de altos ingresos (21-23% vs. 12% respectivamente). En Latinoamérica se estima que hay dos veces más muertes por Enfermedad Cardiovascular (ECV) con un impacto económico de más de 30,9 mil millones de dólares^1^.

En Colombia el comportamiento epidemiológico no ha variado, entre el 2005 y 2016 las enfermedades del sistema circulatorio fueron la primera causa de mortalidad. De acuerdo con el quinto informe del Observatorio Nacional de Salud (ONS), se estima que la enfermedad miocárdica es la más prevalente ocasionando el 53% de las muertes dentro de este grupo^2^.

El impacto de esta enfermedad radica en que, al ser una importante causa de muerte en personas jóvenes, el tiempo de vida laboral disminuye y los gastos de los servicios de salud aumentan, convirtiéndose en un problema de salud pública que requiere de intervenciones inmediatas que contrarresten este aumento^2^. Dentro de las enfermedades cardiovasculares se encuentra la enfermedad isquémica cardiaca, que se produce cuando se reduce la capacidad del músculo cardíaco de bombear sangre, impidiendo el suministro de oxígeno suficiente y generando una sintomatología que pueda ser perceptible o no para el individuo^3^.

Se ha reportado que la persona y su familia después de un evento isquémico cardíaco, experimentan preocupación por el futuro, incertidumbre, miedo a un reinfarto, ansiedad, temor a una muerte inminente, inseguridad para ejercer sus roles normalmente, alteraciones de tipo social, necesidades de apoyo, cambio en el estilo de vida, incapacidad para determinar la causa de los síntomas que pueden aparecer en el tiempo, entre otros. Además, se presenta en muchos casos desconocimiento frente a los factores de riesgo y los síntomas asociados, que pueden llevarle a padecer un infarto agudo de miocardio (IAM), incluso después de haber experimentado la enfermedad^4^.

Estas situaciones pueden impactar sobre la calidad de vida y la adherencia a los tratamientos, por lo que estas preocupaciones deben ser consideradas en las intervenciones en salud por parte de los equipos, con nuevas formas de abordar la educación y el cuidado de las personas. Estas nuevas formas deben contemplar las creencias y perspectivas propias del sujeto de atención y verlo como participante activo y autónomo de su proceso de salud - enfermedad. En este sentido, la comprensión de la apropiación social del conocimiento, como proceso intencionado de interacción e intervención de las relaciones entre ciencia, tecnología y sociedad, que desarrolla nuevas formas de diálogo, mediación y articulación entre actores y sectores desde sus propios roles, saberes y conocimientos es fundamental, ya que permite participar en el diálogo, intercambio, coproducción, comunicación y negociación en torno a la Ciencia, Tecnología e Innovación (CTeI) en donde los ciudadanos toman decisiones mediadas por valores, creencias y formas de ver el mundo se hace cada vez más necesario en la cotidianidad^5^.

Por esta razón, desde la perspectiva de la práctica de la enfermería y la salud pública, el desarrollo de la estrategia de Apropiación Social de la Ciencia, la Tecnología y la Innovación (ASCTeI) que aquí se presenta, busca contribuir al fortalecimiento del cuidado de la salud cardiovascular en las personas con enfermedad isquémica cardiaca, mediante el enfoque de la comunicación social, a través de la herramienta tipo MHealth (página web/app), soportado en el creciente auge en el uso de tecnologías de la información y comunicación (TIC) para la atención en salud, en co-creación con los diferentes actores y articulando la evidencia científica frente a la temática.

## Materiales y Métodos

Estudio de tipo exploratorio que permitió el diseño de una estrategia de apropiación social de conocimiento. La presente estrategia se desarrolló a través de cinco fases que comprenden los principios fundamentales de la ASCTeI:

1. *El reconocimiento del contexto:* hace referencia a la manera de identificar e interpretar la realidad local, sus formas de interacción y convivencia, así como la manifestación de intereses, problemas y necesidades de sus ciudadanos^5^. Para el reconocimiento del contexto se realizó una revisión de la literatura, que permitió evidenciar los avances frente a la enfermedad y su tratamiento.
2. *La participación:* Es comprendida como la intervención ciudadana en asuntos de interés social, que reconoce las interpretaciones y reflexiones del mundo, así como distintos saberes y conocimientos^5^. Para tal fin, se realizaron talleres de co-creación con distintos participantes (entre personas adultas que han sido diagnosticadas con enfermedad isquémica cardiaca, familiares, personal de la salud e investigadores), quienes fueron convocados de manera abierta, mediante la estrategia de bola de nieve, participaron en total 31 personas. Los talleres se realizaron de forma virtual, cada uno con una duración aproximada de 2 horas. Se plantearon cuatro preguntas orientadoras que facilitaron el diálogo y la exploración de las experiencias y conocimientos de los actores involucrados, acerca de las condiciones de salud en la enfermedad isquémica cardiaca.
3. *El diálogo de saberes y conocimiento:* Entendiendo éste como el encuentro entre ciudadanos para intercambiar, compartir y discutir acerca de situaciones de interés, donde se reconocen las diferentes formas de generar y apropiar el conocimiento^5^. Esto se dio en la articulación entre la síntesis de los talleres de co-creación y la evidencia científica sobre la temática que contribuyeron en la creación de una herramienta tipo MHealth.
4. *La confianza:* Es la construcción de relaciones horizontales y transparentes que valoran y reconocen las opiniones^5^. El reconocimiento social de los participantes hacia la Universidad y la práctica de Enfermería permitió establecer un vínculo de confianza frente al proyecto, aspecto que se logra mantener en el tiempo durante las diferentes fases del desarrollo de la estrategia. Frente a los aspectos éticos es importante resaltar que esta estrategia no corresponde a una estructura de metodología de investigación tradicional, por lo que los aspectos éticos contemplados en este proceso están representados en estos principios: el respeto, la veracidad y la autonomía entre los diferentes actores.
5. *La reflexión crítica:* Implica el análisis continuo que hacen los ciudadanos de sus prácticas diarias, situaciones vividas y las condiciones en las que se presentan, con el fin de mejorar y crear nuevas formas de intervenir la realidad. En esta nueva forma de co-crear, surge la reflexión crítica en torno a la construcción de los contenidos de una herramienta tipo Mhealth para el cuidado de la persona con enfermedad isquémica cardiaca. Estas herramientas se consideran como un medio accesible, sencillo y económico, que promueve la continuidad de los cuidados, el automanejo de la enfermedad, facilitan el aprendizaje, el desarrollo de habilidades y mejora la adherencia terapéutica.

La reflexión crítica está representada en la experiencia de construcción y desarrollo de la herramienta tipo MHealth (página web/app) titulada “Cuidando tu corazón” (link de consulta: https://cuidandotucorazon.org/login.html), que permite acceder de manera oportuna a información relacionada con el cuidado cotidiano de la enfermedad isquémica cardiaca y que articula los hallazgos del diálogo de saberes y la evidencia científica reportada en la literatura. Es una app basada en web (WebView) que utiliza lenguajes y librerías de última generación, consolidada, que permite una escalabilidad hacia el futuro. A nivel técnico, es una aplicación creada en el lenguaje de programación PHP, utiliza el sistema de base de datos MariaDB, y para su visualización está basada en el sistema Bootstrap, apoyada por lenguajes como CSS, JavaScript, entre otros.

Es importante mencionar que la información del estudio fue almacenada en Mendeley Data^6^.

Aspectos éticos del estudio: De acuerdo la Resolución 8430 de 1993 el presente estudio fue considerado como un estudio sin riesgo y acato los principios éticos de la declaración de Helsinki para la investigación en seres humanos. Para garantizar la confidencialidad y la autonomía de los participantes se aplicó un consentimiento informado, en el cual el participante podía participar voluntariamente en el estudio y tenía la posibilidad de retirarse en cualquier momento si así lo requería. El estudio contó con aval del Comité de Ética de la Universidad El Bosque (NUR-2021-157).

## Resultados

La estrategia de apropiación social se realizó de manera colaborativa, por medio del diálogo abierto entre las personas con enfermedad isquémica cardiaca, las familias y los profesionales de la salud que participaron en los dos encuentros de co-creación. Los contenidos derivados de los dos talleres fueron organizados y analizados en diagramas de flujo temáticos, que facilitaron la presentación de la información en un lenguaje comprensible para el desarrollo de la herramienta tecnológica.

A continuación, se presentan los resultados en relación al diálogo de saberes y conocimiento, así como el desarrollo de la herramienta tipo Mhealth, que surge de la reflexión crítica como estrategia de apropiación social de conocimiento para el cuidado de la salud cardiovascular en la enfermedad isquémica cardiaca.

### Diálogo de saberes y conocimientos:

El diálogo de saberes y conocimientos es el encuentro entre los distintos actores para intercambiar, compartir y discutir acerca de temas o situaciones de interés común. Se reconoce que el proceso desarrollado en este proyecto a través de los talleres virtuales de co-creación, permitió el abordaje de las experiencias alrededor de la enfermedad isquémica cardiaca, desde la perspectiva del paciente, su familia como cuidadora y los profesionales de la salud. De esta manera, cada uno aportó desde su rol y su propia vivencia el conocimiento propio acerca de la enfermedad, sus necesidades y el cuidado cotidiano alrededor de ella.

Con este ejercicio se identificó que las necesidades de información frente al cuidado, así como las dificultades en la atención en salud, son compartidas por los distintos participantes. El abordaje integral en la atención que incluya todas las dimensiones afectadas, no solo las físicas, sino también las psicosociales e incluso espirituales son necesarias para alcanzar bienestar y calidad de vida después del evento cardiaco. Como hallazgos importantes de este diálogo de saberes y conocimiento se pudo resaltar:

- *Las personas expresaron su experiencia con la enfermedad y las formas de tratamiento recibidas.*
- *Se evidencio poco conocimiento sobre lo que físicamente ocurre en un evento isquémico cardiaco.*
- *Se reconoció la experiencia de la enfermedad isquémica como hecho impactante que modifica el estilo de vida.*
- *Se identifican términos que son utilizados por el equipo de salud que no son claros para la persona y la familia.*
- *La importancia del seguimiento por el equipo de salud no solo en aspectos físicos, sino más de tipo emocional y social.*
- *Los diferentes actores valoraron de manera positiva la información que les permite conocer sobre el proceso de enfermedad y su cuidado.*
- *Las personas dan mayor importancia a los aspectos psicosociales y del entorno al proceso de enfermedad, que, a los aspectos físicos de la enfermedad isquémica cardiaca, que son a los que más énfasis le dan muchos de los profesionales de la salud.*
- *Se reconoce la falta de información sobre cómo abordar el cuidado de la dimensión psicológica e identificación de signos de alarma para buscar ayuda.*
- *Tanto personas, cuidadores y personal de la atención en salud, sienten la necesidad de contar con información más amplia frente a temas de sexualidad, medicamentos, la relación con otros y el apoyo espiritual.*
- *Reconocen la importancia del seguimiento por parte de enfermería; sin embargo, identifican como corta su duración y temporal.*
- *Identifican que la educación durante el proceso de atención en el evento es buena, pero que se requiere mayor profundidad en temas no físicos y a largo plazo.*

Con este primer acercamiento, los participantes reconocieron la importancia de contar con herramientas para el cuidado de la salud elaboradas desde sus propias necesidades, perspectivas, creencias y capacidades, que les permita conocer sobre su propia salud, volverse hábiles en su autocuidado y aceptar la nueva condición de salud adaptándose a un nuevo estilo de vida. Los talleres de co-creación permitieron comprender que las necesidades en cada uno de los actores son distintas, pero a su vez se complementan dando una visión más amplia del proceso de la enfermedad, su tratamiento y la práctica cotidiana del cuidado.

## Reflexión crítica

Con este proceso de apropiación social de conocimiento surgió “Cuidando tu corazón”, una aplicación diseñada y pensada para dispositivos móviles, pero adaptable a todo tipo de pantallas. Al ser pensada para dispositivos móviles, es una aplicación que puede ser usada con una mano, con iconografía estándar y un sistema de colores que permite mantener la experiencia del usuario desde distintos tipos y marcas de dispositivos. La plataforma se escogió debido a su versatilidad y bajo consumo de recursos, lo que permite ser usada en dispositivos de varios años de uso, lo cual tiene en cuenta la facilidad de acceso para la población adulta mayor que puede ser usuaria de la app y que permite su navegación a través de los diferentes recursos.

Posterior al desarrollo de los talleres de co-creación y la construcción conjunta de la herramienta app, se mantuvo seguimiento telefónico con algunos de los participantes, que permitió validar la interpretación de los contenidos indicados por ellos en los talleres para seguir con el montaje de la misma, desde lo temático y los elementos de diseño y desarrollo de la tecnología.

Finalmente, se realizó una validación preliminar con los diferentes participantes en los talleres, quienes navegaron en la herramienta y retroalimentar al grupo desarrollador sobre la aplicación para generar mejoras al producto. Los participantes de manera cualitativa valoraron como positiva la experiencia de participación en la construcción, así como el resultado de la herramienta para el cuidado de la salud cardiovascular en la enfermedad isquémica cardiaca. De esta manera, se contribuye con los procesos de autocuidado para el beneficio de las personas y sus familias, generando una forma accesible, fácil y rápida a información confiable y acorde a sus necesidades, con el fin de mejorar la calidad de vida y el bienestar en personas con isquemia cardiaca.

## Discusión

En la última década ha cobrado importancia la articulación entre los saberes de las comunidades y los resultados de la investigación, con el fin de dar nuevas soluciones a las problemáticas reales y sentidas de la población. De allí que las estrategias de apropiación social sean en la actualidad una herramienta de relevancia e innovadora para la generación de conocimiento en el área de la salud. Sin embargo, dado su auge reciente, aún es escasa la literatura que reporta hallazgos de este tipo de metodologías y su impacto en la práctica cotidiana, especialmente en temáticas de salud cardiovascular que aborden de manera integral a la persona, y que no se encuentren asociadas únicamente a factores de riesgo o de comorbilidades^7^^-^^8^, en la revisión de la literatura efectuada no se encontraron estudios similares que pudieran contrastar o ampliar los hallazgos del presente estudio.

Lo encontrado en el presente proyecto en el diálogo de saberes, reafirma lo reportado en la literatura científica respecto a la información y educación integral que requieren las personas con enfermedad isquémica cardiaca y su familia, que permita abordar los aspectos psicosociales, culturales y espirituales de manera más amplia para garantizar un tratamiento adecuado y que este se mantenga a lo largo del tiempo, sin importar el estrato socioeconómico, el nivel educativo o el tipo de afiliación al sistema de salud. Las personas buscan información y educación que vayan más allá del ámbito físico para contar con habilidades y herramientas que le permitan la reincorporación a la vida cotidiana y afrontar un nuevo estilo de vida transformado por la enfermedad isquémica cardiaca. Los estudios han reportado que la persona y su familia después de un evento isquémico, experimentan preocupación por el futuro, incertidumbre, miedo a un reinfarto, ansiedad, temor a una muerte inminente, inseguridad para ejercer sus roles normalmente, alteraciones de tipo social, necesidades de apoyo, cambio en el estilo de vida, incapacidad para determinar la causa de los síntomas que pueden aparecer posteriormente^9^ situaciones que pueden generar disminución en la calidad de vida e incluso tener un impacto sobre la adherencia a los tratamientos.

Por otra parte, el apoyo social, la identificación de síntomas depresivos, la pérdida del interés, el vivir solos y el estrés financiero se han asociado con un aumento en la mortalidad por cardiopatía coronaria, a pesar del tratamiento médico óptimo de prevención secundaria^10^. También en el caso de los cuidadores, se destacan los cambios en su estilo de vida, donde en algunas ocasiones aprenden por la experiencia, deben estar en constante vigilancia de ser querido, la inversión del tiempo en el cuidado aumenta y como consecuencia pueden presentarse problemas de salud y sobrecarga^11^ Por lo que es importante el abordaje integral de la persona con enfermedad cardiovascular y su familia, lo cual tendrá efectos benéficos para una mejor adaptación psicosocial.

Por otro lado, en los últimos años se ha visto un desarrollo constante en las tecnologías de las telecomunicaciones, con el uso creciente de dispositivos móviles inalámbricos en la población, que permite el acceso a información, incluyendo los temas en salud. Cada vez hay más evidencia que el uso de páginas web y de aplicaciones móviles, además de fomentar el autocuidado y la adherencia, reduce las descompensaciones, los ingresos hospitalarios y las complicaciones en los pacientes crónicos^12^. El paciente y su red de apoyo, entran a formar parte activa en el cuidado de su propia salud y en el tratamiento y seguimiento de su enfermedad; es un ejercicio de autogestión de la enfermedad, de apropiación y de diálogo, que mejora la adherencia al tratamiento con pacientes más informados y, por tanto, más predispuestos a seguir las pautas del tratamiento^13^.

La inclusión de nuevas tecnologías de la información y comunicación (TIC), como las herramientas computacionales e informáticas que procesan, almacenan, sintetizan, recuperan y presentan información, constituyen nuevos soportes y canales para dar forma, registrar, almacenar y difundir contenidos informacionales. Son herramientas y materiales de construcción que facilitan el aprendizaje, el desarrollo de habilidades y distintas formas de aprender. En el ámbito de salud, tecnologías como las aplicaciones móviles han empezado a tomar un lugar importante para pacientes, cuidadores y familias. Su uso puede ser útil para el manejo de condiciones como el de la enfermedad isquémica cardiaca c, ya que permite a los pacientes y su red de apoyo tomar parte activa en el cuidado de su propia salud y acceder a información confiable. Esto mejoraría la adherencia al tratamiento, al contar con pacientes más informados y dispuestos a seguir las pautas del tratamiento, promover la continuidad de los cuidados, el automanejo de la enfermedad, comprender mejor su situación de salud y tratamiento, entre otros. Sin embargo, esto sólo es posible cuando los propios actores pacientes, familias y profesionales de la salud construyen conocimiento conjunto para su diseño y construcción como se plantea en el presente trabajo.

## Conclusiones

La comunicación con enfoque en CTS, es un componente articulador del proceso metodológico, y asume el enfoque del cambio social, entendiendo que el propósito de la ASCTeI es el de aportar a resolver problemas pertinentes de la realidad. Para tal fin, la estrategia de comunicación busca propiciar el diálogo con los diferentes actores para motivar su participación en la gestión del conocimiento, entendiendo que los lenguajes y contextos son diversos y que, por tanto, demandan acciones comunicativas innovadoras y adaptadas.

La construcción de saberes a partir de esta experiencia con este trabajo, mostró que las personas que han padecido una enfermedad isquémica cardiaca, necesitan información sobre diversas temáticas, que contemplan no solo aspectos físicos sino psicosociales e incluso espirituales. Como limitación de este estudio se puede referir el pequeño número de participantes de los diferentes actores del sistema de salud. Esto lleva a pensar en la necesidad de contar con equipos de salud que puedan proporcionar educación integral y abordajes de cuidado que contemplen la experiencia de cada individuo con su enfermedad y una mirada integral a su condición de salud para lograr el éxito terapéutico y las metas en salud con estas personas.
